# Assessment of microstructural abnormalities in gray and white matter of minimal hepatic encephalopathy patients using diffusion kurtosis imaging and their associations with neurocognitive dysfunction

**DOI:** 10.3389/fnhum.2025.1600100

**Published:** 2025-07-18

**Authors:** Qing Sun, Wenliang Fan, Yuan Liu, Zhifeng Kou, Ping Han

**Affiliations:** ^1^Department of Radiology, Union Hospital, Tongji Medical College, Huazhong University of Science and Technology, Wuhan, China; ^2^Hubei Provincial Clinical Research Center for Precision Radiology and Interventional Medicine, Wuhan, China; ^3^Hubei Key Laboratory of Molecular Imaging, Wuhan, China; ^4^Department of Biomedical Engineering, New Jersey Institute of Technology, Newark, NJ, United States

**Keywords:** minimal hepatic encephalopathy, diffusion kurtosis imaging, tract-based spatial statistics, white matter, gray matter, voxel-based morphometry

## Abstract

**Aims:**

Although neural activity abnormalities have been reported in cirrhosis patients with minimal hepatic encephalopathy (MHE), the neurophysiological mechanisms underlying microstructural brain alterations remain poorly understood. This prospective study aimed to assess microstructural abnormalities in both gray matter and white matter of MHE patients by using diffusion kurtosis imaging (DKI), and to examine associations between these alterations and neurocognitive and clinical measurements.

**Methods:**

Thirty-one Hepatitis B Virus-related cirrhotic patients without MHE (NMHE), thirty Hepatitis B Virus-related cirrhotic patients with MHE, and 59 gender-, age-, education-matched healthy controls underwent diffusional kurtosis imaging and neurocognitive assessments. We used tract-based spatial statistics (TBSS) analysis to estimate group differences of white matter (WM) microstructure and voxel-based morphometry analysis to determine gray matter (GM) abnormalities. Correlation analyses were further performed to assess relationships between altered diffusional parameters and clinical variables, such as neurocognitive performances and disease duration.

**Results:**

The TBSS analysis results showed that MHE patients had significantly decreased fractional anisotropy (FA) in the temporal part of the left superior longitudinal fasciculus and decreased kurtosis fractional anisotropy (KFA) in the left corticospinal tract and anterior thalamic radiation (*p* < 0.05, threshold-free cluster enhancement corrected). Notably, lower KFA in WM regions correlated with worse neurocognitive test scores in MHE patients. For GM, MHE patients exhibited increased volume of thalamus. No significant WM or GM differences were observed between NMHE patients and the other two groups.

**Conclusion:**

Minimal hepatic encephalopathy patients demonstrated microstructural abnormalities in both WM and GM, predominantly affecting regions involved in cognitive, attention, and motor functions. These findings suggest that disruption of microstructural integrity may underlie the pathophysiological underpinnings of neurocognitive dysfunction in MHE, offering neuroimaging evidence for disease mechanisms.

## 1 Introduction

Minimal hepatic encephalopathy (MHE) is a common neurological complication of liver cirrhosis, afflicting up to 80% of cirrhotic patients (Acharya and Bajaj, 2018; Dhiman et al., 2010; Maldonado-Garza et al., 2011). However, MHE patients have no recognizable clinical features in the brain, such as neurocognitive impairments. Increasing evidence indicates that MHE patients have a range of subtle neurocognitive dysfunctions (Chen et al., 2021), such as deficits in attention, reduced memory function, abnormal executive performance, impairments in psychomotor speed (Ortiz et al., 2005), visual judgment (Zafiris et al., 2004) and cognitive control (Zhang et al., 2007). Nowadays, MHE has attracted increasing attention because it is closely related to impaired daily functioning (e.g., working disability and impaired driving skill) (Bajaj, 2008; Felipo et al., 2013), decreased quality of life and poor survival (Bajaj et al., 2008). MHE predicts poor prognosis and could represent advanced liver disease. Moreover, patients with MHE fall more often and have a great tendency to progress to overt hepatic encephalopathy (HE), which is a more serious complication of cirrhosis with significant mortality and obvious clinical neurocognitive deficits. The early diagnosis of MHE is critical for timely treatment and improving the prognosis of MHE patients (Bajaj et al., 2012). However, MHE-related neurocognitive impairments often evade clinical detection due to their subtle and non-specific presentation. Moreover, the pathophysiological mechanisms responsible for these neurocognitive deficits remain elusive.

Most prior magnetic resonance imaging (MRI) studies of MHE have relied on conventional diffusion tensor imaging (DTI) to assess white matter (WM) changes (Chen et al., 2021; Mei et al., 2023; Qi et al., 2013). However, emerging neuroimaging evidence suggests that DTI assumes that diffusion in all directions is Gaussian, which may capture WM fiber loss-related extracellular diffusivity but fails to fully reflect myelin sheath complexity and cellular compartment changes in intact fibers. In contrast to conventional DTI, diffusion kurtosis imaging (DKI) can quantify water mobility direction and tissue complexity, offering a more comprehensive assessment of WM microstructural alterations beyond macrostructural neuroimaging (Chen et al., 2012c; Jensen et al., 2005). By characterizing non-Gaussian diffusion behavior in neural tissues, DKI demonstrates exceptional sensitivity in detecting developmental and pathological changes in brain microstructure [e.g., in aging (Falangola et al., 2008)], and quantifies WM complexity and compartmentalization beyond conventional DTI (Steven et al., 2014). It is reported that the DKI-derived estimates of the diffusion parameters are generally more accurate and sensitive than those same metrics obtained from conventional DTI (Ito et al., 2017; Jensen et al., 2005; Taha et al., 2022). Kurtosis information may show early microstructural changes in some diseases before these morphologic changes are seen with conventional MRI techniques (Steven et al., 2014). Therefore, DKI may provide new insights in the pathogenesis of MHE and may be helpful for investigating abnormalities in tissues with isotropic microstructure in which DTI are less useful.

The tract-based spatial statistics (TBSS) analysis, an automated, observer-independent method, is a widely used tool for the analysis of diffusion MRI in white matter (Asaf et al., 2015; Giannakis et al., 2024; Smith et al., 2006). It has already been validated as an effective way to evaluate the diffusional metrics in many diseases (Asaf et al., 2015; Chylinska et al., 2022). However, no prior study has performed TBSS analysis of DKI data to assess microstructure abnormalities in one cohort HBV patients with cirrhosis and MHE and their relationships with clinical variables. Additionally, given that gray matter (GM) and white matter collectively form the structural basis of the brain, MRI-based GM studies are critical for unraveling the pathophysiology of MHE (Iwasa et al., 2012; Qi et al., 2012; Qi et al., 2013). Voxel-based morphometry (VBM), a versatile analytical technique, can quantify voxel-level volumetric differences in brain tissues, including GM. Thus, this study applied this technique to investigate changes in GM volume in cirrhotic patients.

To date, MHE remains a poorly explored field, with its neuropathological mechanisms and their association with neurocognitive abnormalities still lacking comprehensive characterization. Notably, certain medications (e.g., Babao Dan, benzodiazepines) used in the management of MHE have been reported to influence neurocognitive function (Lee et al., 2014; Lu et al., 2021). To control for potential medication-related confounds, we excluded participants who were receiving these pharmacological treatments. In this prospective study, we hypothesized that microstructural abnormalities in the white matter and gray matter of MHE and/or NMHE patients with cirrhosis would be identified through TBSS analysis of DKI data and VBM analysis of T1-weighted imaging. In addition, we hypothesized that these abnormalities would exhibit significant correlations with clinical parameters, such as neurocognitive performances.

## 2 Materials and methods

### 2.1 Participants

This study was approved by the medical ethics committee of Tongji Medical College of the Huazhong University of Science and Technology. Written informed consent was obtained from each participant prior to involvement in this study.

All subjects were given standard neurocognitive tests: the mini-mental status examination (MMSE) and the five test psychometric hepatic encephalopathy score (PHES) (Lv et al., 2013; Weissenborn, 2008), including number connection test A (NCT-A), number connection test B (NCT-B), the digit-symbol test (DST), serial dotting test (SDT), and line tracing test (LTT). A neurologists was blind to subjects’ healthy statues and identity and gave neurocognitive tests scores. The subjects were only allowed to proceed if their MMSE result exceeded 25, to eliminate patients with overt HE. The PHES battery has been widely used to diagnose MHE (Weissenborn, 2008) and evaluates many neurocognitive functions such as motor speed, visual, memory, attention functioning, and concentration (Weissenborn, 2008). The method for calculating PHES scores has been described in detail in a previous study (Lv et al., 2013). In the NCT-A, NCT-B, SDT and LTT tests, a longer completion time represents worse performance, whereas in DST, a lower score represents worse performance. Cirrhotic patients were diagnosed with MHE if they showed no clinical symptoms of overt HE and had abnormal scores on the PHES test, defined as at least two of the PHES battery tests beyond two standard deviations (SDs) of the mean value for the age/gender/education-matched healthy controls (Lv et al., 2013). All subjects underwent brain MRI after they finished all neurocognitive tests.

This prospective study enrolled thirty hospitalized right-handed cirrhotic patients with MHE, thirty-one hospitalized right-handed cirrhotic patients without MHE, and fifty-nine healthy controls matched for age, gender, and education level. The mean disease duration was 8.7 years in MHE patients and 5.5 years in NMHE patients. Both groups of patients received basic antiviral treatment, mainly using nucleos(t)ide analogs such as entecavir, tenofovir disoproxil fumarate, and tenofovir alafenamide to inhibit hepatitis B virus replication. Twenty-four cirrhotic patients took liver-protective/antifibrotic agents (e.g., Chinese herbal medicines). Additionally, 38 cirrhotic patients had a history of or were currently taking medications for the treatment of complications, such as using diuretics like spironolactone and furosemide to control ascites, or somatostatin analogs and β/α-blockers to control and/or prevent variceal bleeding. Most patients had a history of or were currently taking probiotics to modulate gut microbiota. None of the healthy participants suffered from any types of hepatitis, cirrhosis, or neurological/psychiatric diseases. Each control had normal cognitive function and brain MRI imaging results.

The inclusion criteria for patient recruitment were as follows: had clinically proven hepatitis B virus-related cirrhosis based on their medical history, clinical examination or biochemical and medical imaging findings, such as abdominal computerized tomography and ultrasound examination; no other types of cirrhosis; without clinical manifestation of overt hepatic encephalopathy (HE); could finish the MRI examination without any MRI contraindication; right-handedness. The liver functional status of each cirrhotic patient was assessed according to the Child-Pugh scores.

Exclusion criteria for all patients and controls included current overt HE or history of overt HE, any contraindication to MRI, any obvious brain lesions (such as brain contusion or tumor), carcinoma, severe history of medical problems (such as congestive heart failure), neurological or psychiatric disorders, taking some medicine (e.g., Babao Dan, benzodiazepines) affecting cognitive function, severe organic diseases (such as kidney failure), or alcohol abuse in the 6 months before the study, left-handedness.

### 2.2 MRI techniques

Experiments were conducted on a 3-Tesla GE Discovery MR 750W (Grandview Blvd Waukesha, WI, United States) with an eight-channel phased-array head coil. Each subject was scanned in a head-first position with symmetrically placed cushions on both sides of the head to decrease motion.

The DKI experiments were performed using the diffusion sequence along 30 different diffusion encoding directions. The imaging parameters were as follows: repetition time (TR) = 8,000 m, echo time (TE) = minimum, field of view (FOV) = 25.6 × 25.6 cm^2^, matrix = 128 × 128, slice thickness = 4.0 mm, three b-values (b = 0, 1,000, and 2,000 s/mm^2^), and flip angle = 90°. The entire process took 8 min and 16 s to complete. T1-weighted images were acquired using a three-dimensional brain volume imaging (3D BRAVO) sequence with parameters covering the whole brain (TR/TE = 9.1/400 ms, slice thickness = 1.1 mm, gap = 0 mm, matrix = 256 × 256, FOV = 25.6 × 25.6 cm^2^, and acquisition time = 4 min and 54 s). We also collected T2-weighted fluid-attenuated inversion recovery (FLAIR) images (TR/TE = 12,000/120 ms, slice thickness = 4.0 mm, gap = 0 mm, matrix = 320 × 224, and FOV = 24 × 21.6 cm^2^). 3D BRAVO and FLAIR sequences were employed to exclude anatomic and pathological abnormalities from consideration. Additionally, the T2-star weighted angiography sequence were performed to exclude intracranial blood vessels abnormalities: FOV = 24.0 × 24.0 cm^2^; slice thickness = 4.0 mm, TR = minimum; TE = 24.6 ms; flip angle = 20°. Two experienced radiologists were responsible for ensuring high-quality MRI images and excluding those subjects who had brain structure abnormalities on routine MRI.

### 2.3 Image analysis

Experimental flow chart can be found in Supplementary Figure 1.

#### 2.3.1 TBSS analysis of DKI data

The TBSS allows for group-wise comparisons of DKI data by projecting fractional anisotropy (FA)/kurtosis fractional anisotropy (KFA) data from each subject onto a voxel skeleton located at the center of major cerebral white matter pathways through the whole brain, thereby minimizing the registration error compared to voxel-based analysis using statistical parametric mapping and minimizing the personal evaluation bias compared to region-of-interest-based analysis reported in prior diffusion MRI studies (Asaf et al., 2015).

Diffusion kurtosis imaging analysis was performed using Functional MRI of the Brain (FMRIB) Software Library (FSL)^1^ and Diffusional Kurtosis Estimator (DKE) version 2.6.0^2^ (Narita et al., 2016). After the eddy current correction by using affine alignment of each image to the b0 image, the DKI data were then processed using DKE software to generate maps of DKI metrics [i.e., KFA and mean kurtosis (MK)] and DTI metrics [i.e., FA and mean mean diffusivity (MD)] (Narita et al., 2016). Then, whole-brain voxel-wise differences between MHE patients and healthy controls were evaluated using the TBSS toolbox of FSL via the following four steps. First, FA, MD, KFA and MK images of all subjects were transformed into 2 × 2 × 2 mm^3^ Montreal Neurological Institute 152 (MNI152) common space via non-linear registration. Second, the transformed FA and KFA images were averaged to create mean FA and KFA images and thinned to generate mean FA and KFA skeletons, respectively. Then, each subject’s aligned FA and KFA maps were mapped onto the “mean FA and KFA skeleton” using a threshold of 0.2 to exclude the gray matter. The method of tuned non-linear registration, followed by the generation of the mean FA and KFA skeletons, aims to overcome the potential cross-subject spatial variability effect. For each participant, registered FA and MD maps were projected onto the FA skeleton while registered KFA and MK maps were projected onto the KFA skeleton. Then the resulting data were inputted into voxel-wise cross-subject statistics.

#### 2.3.2 Voxel-based morphometry (VBM) analysis

To further investigate the gray matter (GM) abnormalities and its relationship with patients’ neurocognitive performances, we performed VBM analysis. VBM is considered as a fully automated and unbiased imaging analysis technique for characterizing regional cerebral gray volumes (Iwasa et al., 2012). It was used to map the statistical probability of differences in regional tissue volume between diagnostic groups. The 3D BRAVO images were processed using in Statistical Parametric Mapping (SPM12)^3^. These structural images were corrected for bias-field inhomogeneity, registered using linear (12-parameter affine) and non-linear transformations, and segmented. Then, the T1-weighted template, white matter, gray matter and cerebrospinal fluid probability maps were obtained by averaging across subject data in the MNI space. All native space gray matter images were registered to the template, spatially normalized into standards MNI space, and modulated for non-linear components. Finally, the resulting gray matter images were smoothed with an 8-mm full-width at half maximum (FWHM) Gaussian kernel before their use as input for the statistical analysis.

### 2.4 Statistical analysis

The one-way analysis of variance (ANOVA) and Tukey’s post-hoc test were applied to compare group differences in the age, education level and neurocognitive test scores among MHE patients, NMHE patients, and healthy subjects. The Chi-square test was used to compare Child-Pugh classification and gender distributions across groups. Differences in MMSE scores among these groups were evaluated using a Kruskal-Wallis test. Two-tailed independent samples *t*-test was used to examine the differences in the disease duration, diameters of the portal vein and splenic vein. All analyses were performed using the Statistical Package for Social Sciences (SPSS, version 20; IBM Corporation, United States), and the results are given as the mean ± SD. A *p*-value < 0.05 was deemed statistically significant.

#### 2.4.1 TBSS analysis

For voxel-wise statistics in TBSS analysis of these diffusion and kurtosis metrics, the randomized tool, i.e., a permutation-based inference tool, was applied in FSL to compare the skeletonized FA, MD, KFA and MK maps among three groups (NMHE patients, MHE patients, and healthy controls) with adjustments for all participants’ age and gender, and the number of permutations was set to 5,000. Threshold-free cluster enhancement (TFCE) (Qi et al., 2013) was used to obtain the significant group differences among these groups. The significance threshold for between-group differences was set at *p* < 0.05 after accounting for multiple comparisons by controlling for family-wise error rate (FWE). The aberrant diffusion and kurtosis metrics were extracted from brain clusters with significant differences for further analysis.

#### 2.4.2 VBM analysis

To examine group differences in gray matter volume, one-way analysis of variance and *post hoc* two-sample tests were used to detect differences in gray matter volume among MHE patients, NMHE patients, and healthy controls. The statistic threshold was set at false discovery rate (FDR) corrected *p* < 0.0001, with a cluster size of at least 200 voxels. The clusters with significant differences were extracted.

#### 2.4.3 Correlation analysis

The correlation analysis was performed to investigate the relationships between both abnormal diffusion and kurtosis metrics and gray matter volume and clinical variables (e.g., disease duration and neurocognitive test scores) using SPSS 18.0 software (SPSS, Inc., Chicago, IL, United States). If the data were fit into a normal distribution, the correlation analysis used the Pearson correlation analysis, otherwise, the Spearman correlation analysis was performed. A value of *p* < 0.05 was considered statistically significant.

## 3 Results

### 3.1 Demographic and neurocognitive characteristics

Table 1 shows the demographic and clinical characteristics of all subjects. ANOVA and post-hoc tests showed no significant between-group differences among the three groups in terms of the age, gender and education level in this study (all *p* > 0.05). There was significant between-group differences in disease duration and Child-Pugh classification between MHE patients and NMHE patients (*p* < 0.05). The MMSE scores of the three groups were normal, and no significant differences were observed among these groups (*p* > 0.05). ANOVA test revealed significant inter-group differences among the three groups (NMHE patients, MHE patients, and healthy controls) (*p* < 0.05). The results of Tukey’s post-hoc test showed that cirrhotic patients with MHE had significantly worse neurocognitive test scores (i.e., PHES, NCT-A, NCT-B, SDT, DST, LTT) as compared with healthy subjects/NMHE patients (*p* < 0.05). No significant difference was observed in all neurocognitive test scores between NMHE patients and healthy controls (all *p* > 0.05).

**TABLE 1:** Demographic and clinical characteristics of MHE and NMHE patients, and healthy subjects.

Demographic	MHE (*n* = 30)	NMHE (*n* = 31)	Control (*n* = 59)	*P*-value
Gender (male/female)	23/7	27/4	44/15	*P* = 0.379
Age (years)	47.8 ± 11.1	46.4 ± 8.3	46.0 ± 9.9	*P* = 0.714
Education level (years)	6.7 ± 4.4	7.6 ± 4.8	8.4 ± 3.6	*P* = 0.177
Disease duration (years)	8.7 ± 4.8	5.5 ± 3.9	–	*P* = 0.007*
Child–Pugh’s class: A/B/C (*n*)	8/10/12	22/3/6	–	*P* = 0.002*
MMSE (scores)	27.5 ± 1.4	27.7 ± 1.3	28.1 ± 1.3	*P* = 0.145
PHES (scores)	−7.6 ± 2.9	0.3 ± 1.0	0.1 ± 1.5	*P* < 0.001*
DST (scores)	32.6 ± 5.9	49.5 ± 9.5	49.5 ± 9.9	*P* < 0.001*
SDT (scores)	64.9 ± 10.4	49.4 ± 6.0	44.7 ± 8.5	*P* < 0.001*
NCT-A (scores)	70.9 ± 12.4	52.1 ± 8.0	46.4 ± 10.1	*P* < 0.001*
NCT-B (scores)	96.4 ± 16.7	65.0 ± 10.5	59.7 ± 12.2	*P* < 0.001*
LTT (scores)	70.6 ± 11.2	52.9 ± 6.2	47.5 ± 9.2	*P* < 0.001*
Portal vein diameter (mm)	15.6 ± 1.3	13.8 ± 1.1	–	*P* < 0.001*
Splenic vein diameter (mm)	12.9 ± 1.3	10.7 ± 1.3	–	*P* < 0.001*

*Significant differences (*p* < 0.05). All values are displayed as mean ± standard deviation (SD). The *p*-values for gender distribution and Child–Pugh classification were obtained by chi-square test. The Kruskal-Wallis test was utilized to compare MMSE scores. Two-tailed independent samples *t*-test was used to examine the differences in the disease duration, diameters of the portal vein and splenic vein. The *p*-values for age, education level and neurocognitive test scores were obtained by one-way analysis of variance. MHE, minimal hepatic encephalopathy; NMHE, without minimal hepatic encephalopathy; MMSE, Mini-Mental State Examination; PHES, psychometric hepatic encephalopathy score; DST, digit-symbol test; SDT, serial dotting test; NCT-A, the number connection test-A; NCT-B, number connection test B, LTT, line tracing test.

### 3.2 TBSS analysis of group differences in the parameters

**FIGURE 1:**
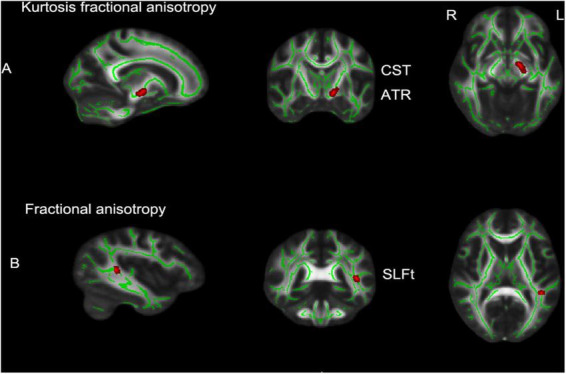
TBSS analytical results in patients with minimal hepatic encephalopathy compared with the control group (*p* < 0.05, TFCE corrected, 5,000 permutations). **(A)** The brain regions with significantly decreased kurtosis fractional anisotropy (KFA) values in left CST and left ATR; **(B)** decreased fractional anisotropy (FA) values in the SLFt of minimal hepatic encephalopathy (MHE) patients compared to healthy controls. Rows show selected sagittal, coronal and axial maxima coordinate slices on a Montreal Neurological Institute 152 (MNI152) brain template image (MNI coordinates). Red voxels indicate significantly altered values. TBSS, tract-based spatial statistics; L, left; R, right; CST, corticospinal tract; ATR, anterior thalamic radiation; SLFt, temporal part of the left superior longitudinal fasciculus; TFCE, threshold-free cluster enhancement.

The brain regions with significant differences between the MHE group and healthy controls are shown in Figure 1 and listed in Table 2. Decreased KFA values were observed in the left corticospinal tract (CST) and left anterior thalamic radiation (ATR) in the MHE group when compared with healthy subjects (Figure 1A) (*p* < 0.05, TFCE corrected). FA reductions were found in the temporal part of the left superior longitudinal fasciculus (SLF) in MHE patients compared to healthy controls (Figure 1B) (*p* < 0.05, TFCE corrected). There was no significant difference in MD and MK metrics among the three groups. Group comparisons showed no significant inter-group differences in DKI parameters in NMHE patients relative to the other two cohorts.

**TABLE 2:** Locations and sizes of brain voxel clusters with decreased kurtosis fractional anisotropy (KFA) and fractional anisotropy (FA) values from minimal hepatic encephalopathy patients compared with those of healthy controls (*p* < 0.05, TFCE corrected, 5,000 permutations).

Diffusional metrics	Anatomic regions	Peak MNI coordinates	*P*-value
		**X Y Z**	
Kurtosis fractional anisotropy	Corticospinal tract_L Anterior Thalamic Radiation_L	−12 −9 −9	0.045
Fractional anisotropy	Superior longitudinal fasciculus_L (in the temporal part)	−42 −41 9	0.025

MNI, Montreal Neurological Institute; TFCE, threshold-free cluster enhancement; L, left; MNI, Montreal Neurological Institute.

### 3.3 Differences in regional gray matter increase between groups

**FIGURE 2:**
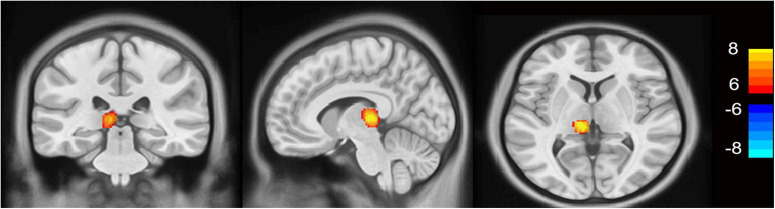
VBM results in patients with minimal hepatic encephalopathy compared to healthy controls (*p* < 0.0001, FDR corrected). The minimal hepatic encephalopathy (MHE) group had significantly higher thalamic gray matter volume than the control group (FDR *p* < 0.0001 corrected for multiple comparisons, with a cluster size of at least 200 voxels). Yellow voxels indicate significantly increased values. VBM, voxel-based morphometry; FDR, false discovery rate; L, left; R, right.

As shown in Figure 2, the MHE group had significantly higher thalamic gray matter volume than control group (FDR *p* < 0.0001 corrected for multiple comparisons, with a cluster size of at least 200 voxels). No statistically significant between-group differences were detected in the gray matter volume of NMHE patients compared to the other two groups.

### 3.4 Correlations between clinical indicators and white and gray matters variables

**FIGURE 3:**
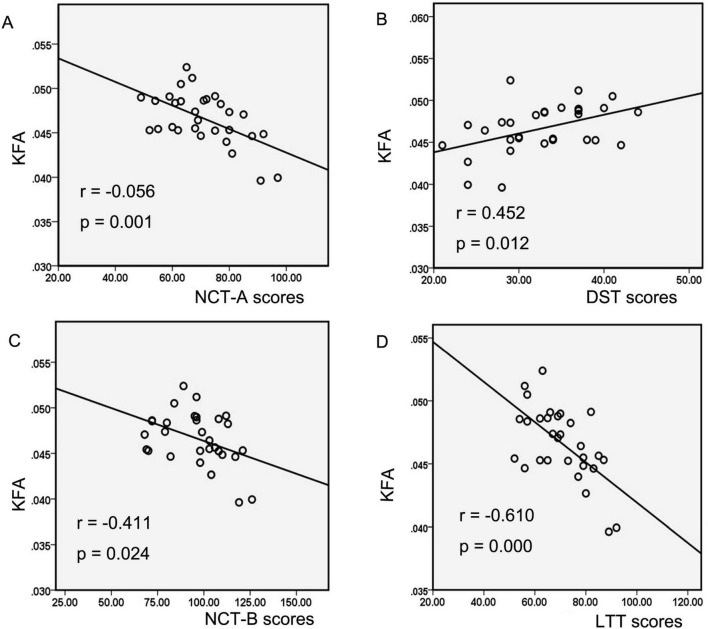
The correlations between diffusion and kurtosis parameters and neurocognitive performances in MHE patients. Correlation analysis showed decreased KFA values of the left CST and ATR in the MHE group were negatively correlated with the NCT-A scores **(A)**, NCT-B scores **(B)**, and LTT scores **(C)**, as well as positively correlated with the DST scores **(D)**. MHE, minimal hepatic encephalopathy; KFA, kurtosis fractional anisotropy; CST, corticospinal tract; ATR, anterior thalamic radiation; NCT-A, number connection test-A; NCT-B, number connection test B; LTT, line tracing test; DST, digit-symbol test.

Correlation analysis explicitly showed significant negative correlations between decreased KFA values in the left CST and left ATR and neurocognitive test scores in the MHE group, including NCT-A scores (negative correlation, significant, r = −0.056, *p* = 0.001; Figure 3A), NCT-B scores (negative correlation, significant, r = −0.411, *p* = 0.024; Figure 3B), and LTT scores (negative correlation, highly significant, r = −0.610, *p* = 0.000; Figure 3C). Additionally, a significant positive correlation was observed between decreased KFA values and DST scores (positive correlation, significant, r = 0.452, *p* = 0.012; Figure 3D). In MHE patients, neither the abnormal KFA/FA values nor increased thalamic volume correlated significantly with disease duration or portal/splenic vein diameters (all *p* > 0.05). Furthermore, thalamic volume changes showed no significant association with neurocognitive test scores in this cohort (all *p* > 0.05).

## 4 Discussion

To the best of our knowledge, no prior study has performed TBSS analysis of DKI data to assess microstructure abnormalities in one cohort Hepatitis B virus-related cirrhosis with MHE. In the present study, we investigated both whole-brain gray matter (GM) volume and white matter (WM) microstructure integrity in cirrhotic patients with or without MHE. In WM, the TBSS analysis exhibited reduced fractional anisotropy (FA) and kurtosis fractional anisotropy (KFA) in WM tracts in MHE patients compared with the control group. Notably, decreased KFA values significantly correlated with poorer neurocognitive performances, suggesting a link between WM microstructure and cognitive dysfunction in MHE patients. In GM, MHE patients demonstrated increased thalamic volume compared with healthy controls.

### 4.1 White matter abnormalities

Our results of significantly decreased FA and KFA regions, i.e., left corticospinal tract (CST), left anterior thalamic radiation (ATR) and left superior longitudinal fasciculus (SLF), in MHE patients are consistent with previous neuroimaging findings from diffusion MRI studies in cirrhosis with MHE (Chen et al., 2012a; Gomez-Anson et al., 2015; Gupta et al., 2021; Montoliu et al., 2014; Qi et al., 2013). These findings indicated white matter abnormalities in MHE patients. The FA parameter measures diffusion fractional anisotropy, and reflects the directional properties of water molecules in a magnetic field, associating with the integrity of myelinated fibers and the density of white matter nerve fibers. When there are changes in the microstructural integrity, the FA parameter decreases (Li et al., 2024; Xu et al., 2023). The KFA has a pure property of the kurtosis tensor, and can measure anisotropy. It uses the fourth-order kurtosis tensor for calculation, making it less vulnerable to errors from complex WM fibers such as crossing fibers and more useful for evaluating tissue complexity than conventional DTI-derived metrics (Veraart et al., 2011). A decrease in KFA parameter of white matter fiber bundles suggests a reduction in complexity and weakening of non-linear features. Moreover, it is reported that FA and KFA can complement each other to reflect the complexity of cytoskeletal architecture and microstructural integrity of brain tissue in some extent (Bester et al., 2015; Glenn et al., 2015).

#### 4.1.1 FA abnormalities in the SLF fiber tract

Our findings of white matter regions with decreased FA in the SLF as compared with control subjects were in accordance with some previous DTI studies (Chen et al., 2021; Montoliu et al., 2014; Qi et al., 2013). FA reflects myelin integrity. The lower white matter integrity, the lower the FA value. Decreased FA has been considered as associated with the disruption of brain intrinsic networks, which can induce neurologic dysfunction in MHE patients (Lin et al., 2012; Qi et al., 2012). The SLF, which contains a comparatively high number of multi-fiber orientations, is considered to be the largest associative fiber bundle system in the brain. It is a part of the longitudinal association fiber system, which lays connections between the frontal lobe and other areas, such as temporal and parietal (Ayyıldız et al., 2023; Janelle et al., 2022). The SLF pathway has been demonstrated to be associated with visuospatial attention and memory decline (Ayyıldız et al., 2023; Klarborg et al., 2013; Tullberg et al., 2004). As reported previously, attention and cognitive deficits have been considered as features of MHE (Felipo et al., 2012; Weissenborn et al., 2001). Therefore, we speculate that local white matter changes in attention-related regions (i.e., SLF) may reflect clinical manifestations in MHE patients, such as distractibility.

#### 4.1.2 Group differences in the KFA

We found brain regions with significantly decreased KFA in the left CST and ATR in the MHE group, which paralleled one previous study (Gupta et al., 2021), highlighting impaired directional coherences of the brain microstructures in MHE patients. The CST is a descending motor pathway that is mainly involved in body movement control (Bang et al., 2015). As reported previously, some atypical behavioral symptoms of MHE, such as falling easily, may result from aberrations in motor-related white matter microstructure (Gomez-Anson et al., 2015). Additionally, the PHES battery test can recognize features of alterations in neuronal coordination of MHE patients, especially cognitive and psychomotor domains (Maldonado-Garza et al., 2011). The correlation between the decreased KFA in the CST and poor neurocognitive performances of MHE patients are reasonable. Moreover, damages in the white matter integrity of the CST may affect neurotransmitters’ neurotransmission, especially glutamate, which is the main neurotransmitter in the CST. Abnormal glutamatergic neurotransmission has been revealed to be involved in hepatic encephalopathy (Butterworth, 2000); thus, we speculate that this alteration may be associated with neuropathological changes in MHE. Regarding the ATR, the white matter of the thalamic radiation links the frontal cortex with the thalamus, and reciprocal interactions between the two regions play an essential role in cognition (Collins et al., 2018). The ATR is mainly involved in executive functioning, memory encoding and attention control (Ayyıldız et al., 2023; Mamah et al., 2010; Van der Werf et al., 2003; Zou et al., 2008). From a neuropsychological perspective, a cognitive-behavioral dysfunction may result from white matter subcortical lesions since they disrupt interconnections between brain regions in distributed neural networks. Therefore, decreased KFA in ATR in MHE may be related to abnormalities in the cognition and attention functions, reflected by poor neurocognitive performances. This finding was similar to one study by Montoliu et al. (2014), in which they also detected reduced white matter micro-integrity in the left ATR in MHE.

Furthermore, decreased KFA in these regions where was associated with neurocognitive test results of MHE patients. Similar to our findings, Chen et al. (2012a; 2021) and Montoliu et al. (2014) also found a correlation between poor neurocognitive performances and WH alterations using DTI. The correlations between decreased KFA value and neurocognitive test scores of MHE patients further suggest that WM abnormalities, especially in the left CST and ATR, are responsible for neurologic deficits such as psychomotor, attention and visual memory, and visuospatial motor function. Our findings indicate a correlation between microstructual changes in these brain regions and subclinical neurocognitive damages in MHE, and the KFA is a sensitive parameter to identify MHE patients. Therefore, our study provided more insights in the pathophysiology of MHE.

The observed reductions in FA and KFA values in white matter strongly suggest microstructural integrity disruption in MHE patients. These diffusion abnormalities likely reflect irreversible neuropathological changes, as supported by previous autopsy studies demonstrating axonal loss and demyelination in chronic hepatic encephalopathy patients with corresponding white matter lesions (Chavarria et al., 2011; Guevara et al., 2011; Matsusue et al., 2005). These white matter alterations may result from multiple interrelated pathophysiological mechanisms, including neuroinflammatory processes (oxidative stress and neuroinflammation leading to oligodendrocyte apoptosis and demyelination) (Atluri et al., 2011), cytotoxic edema from metabolic disturbances, cytoskeletal alterations due to neurotoxic metabolite accumulation, and gliopathy involving astrocytic and microglial dysfunction (McTigue and Tripathi, 2008).

#### 4.1.3 Quantitative measurements in the mean diffusivity (MD) and mean kurtosis (MK)

Mean diffusivity reflects alterations in membrane or other barriers to water diffusion; MK reflects tissue microstructural complexity. MD/MK values showed no significant differences among the three groups (MHE patients, NMHE patients, and healthy controls). These results weren’t in agreement with several previous studies, which reported an increase/decrease in MD and decreased MK, when comparing MHE patients with the controls (Chavarria et al., 2011; Chen et al., 2017; Qi et al., 2013). Chavarria et al. (2011) used biexponential analysis of DTI data to analyze cirrhotic patients and found increased MD. These divergent findings likely reflect methodological variations, particularly in the choice of diffusion imaging sequences and post-processing approaches, which are known to influence quantitative diffusion metrics. One prior study reported lower MK in cirrhotic patients without overt hepatic encephalopathy compared with the control group (Chen et al., 2017). The discrepancy with our findings may be attributed to two key factors: (1) their lack of MHE/NMHE stratification, potentially obscuring subgroup-specific effects; and (2) the study’s small sample size of only eighteen cirrhotic patients, which likely resulted in insufficient statistical power to detect subtle differences. Moreover, previous MR diffusion studies in chronic cirrhotic patients have reported abnormal MD values (Chavarria et al., 2011; Kale et al., 2006), with these abnormalities reversing to normal following effective therapy (Poveda et al., 2010) or liver transplantation (Chavarria et al., 2011). We speculate that the unchanged MD/MK values in this study may be attributed to either the disease not yet causing alterations in these parameters or effective treatment normalizing them. Subsequent investigations will be undertaken to substantiate our hypotheses.

### 4.2 Increased gray matter volume

To investigate potential gray matter contributions to neurocognitive impairments, we conducted voxel-based morphometry (VBM) to examine gray matter volumetric alterations. Our results showed significantly increased GM volume in the left thalamus in patients with MHE compared with healthy controls. This finding was similar to previous studies (Qi et al., 2013; Tao et al., 2013). Tao et al. (2013), revealed increased volume in thalamus in patients with MHE/overt HE. Chen et al. (2012b), found increased thalamic volume in 21 cirrhotic patients with a history of overt HE. However, García-García et al. (2017) indicated that MHE patients had reduced GM in the right frontal lobe, insula, and cerebellum compared to NMHE patients, which were inconsistent with our results. This discrepancy may be related to their study’s smaller sample size (13 MHE patients) and etiological heterogeneity including alcoholic cirrhosis. Given that the thalamus plays a crucial role in for the cortical–striatum–thalamic circuit, which is associated with mediating emotional, emotional drive and cognitive processing (Haber and Calzavara, 2009; Tao et al., 2013). Increased thalamic volume in the MHE patients may suggest neuronal and/or glial hypertrophy or hyperplasia, and has been believed as a compensatory effect.

In short, we identified abnormalities in the WM pathways and increased thalamic volume in MHE patients. Decreased FA and KFA observed in MHE might be interpreted as an effect caused by impairments in microstructure integrity of specific white matter tracts. Considering that these abnormal brain regions (i.e., SFG, ATR, and CST) are mainly involved in attention, cognition, and motor functions, we speculate that these findings may explain the neurocognitive and psychomotor impairments experienced by MHE patients. The correlations between the kurtosis metric (i.e., KFA) and poor neurocognitive test performances in MHE further confirmed our speculation.

### 4.3 Limitations and future work

This study has several limitations. First, the sample size in the present study was relatively small; thus, it is possible that the analyses are under-powered to find alterations in MD and MK metrics. Second, the cross-sectional nature of this study limits our ability to monitor the evolution of brain microstructural changes on an individual basis. Third, we did not include cirrhotic patients with overt HE because most cirrhotic patients with overt HE would not be able to remain static during our MRI imaging due to their serious clinical conditions and poor compliance. Therefore, in the future, longitudinal studies should be performed to verify the results and investigate the dynamic changes of brain micro-structure by following a larger homogeneous patient cohort over a longer time period.

## 5 Conclusion

In conclusion, we identified distinct microstructural abnormalities in both white and gray matter of MHE patients compared with healthy controls. These findings indicate that such microstructural disruptions may underlie the neurocognitive and motor impairments characteristic of MHE, offering novel neuroimaging insights into its pathophysiology.

## Data Availability

The original contributions presented in the study are included in the article/Supplementary material, further inquiries can be directed to the corresponding author/s.

## Footnotes

1
http://www.fmrib.ox.ac.uk/fsl

2
http://nitrc.org/projects/dke

3
http://www.fil.ion.ucl.ac.uk/spm/

## Supplementary material

Supplementary Figure 1 Experimental flowchart. DKI, diffusion kurtosis imaging; BRAVO, three-dimensional brain volume imaging; DKE, Diffusional Kurtosis Estimator; TBSS, tract-based spatial statistics; VBM, voxel-based morphometry; FA, fractional anisotropy; KFA, kurtosis fractional anisotropy; MK, mean kurtosis, MD, mean diffusivity; MNI152, Montreal Neurological Institute 152; TFCE, Threshold-free cluster enhancement.
